# Differential Effect of Active Smoking on Gene Expression in Male and Female Smokers

**DOI:** 10.4172/2157-2518.1000198

**Published:** 2014-10-31

**Authors:** Sunirmal Paul, Sally A Amundson

**Affiliations:** 1 Center for Radiological Research, Columbia University Medical Center, New York, NY; 2 Department of Radiology, New Jersey Medical School, Cancer Center RUTGERS, Newark, NJ

**Keywords:** Smoking and sex, Smoking and immune response, Smoking carcinogenesis, Gene expression analyses, Microarray

## Abstract

Smoking is the second leading cause of preventable death in the United States. Cohort epidemiological studies have demonstrated that women are more vulnerable to cigarette-smoking induced diseases than their male counterparts, however, the molecular basis of these differences has remained unknown. In this study, we explored if there were differences in the gene expression patterns between male and female smokers, and how these patterns might reflect different sex-specific responses to the stress of smoking. Using whole genome microarray gene expression profiling, we found that a substantial number of oxidant related genes were expressed in both male and female smokers, however, smoking-responsive genes did indeed differ greatly between male and female smokers. Gene set enrichment analysis (GSEA) against reference oncogenic signature gene sets identified a large number of oncogenic pathway gene-sets that were significantly altered in female smokers compared to male smokers. In addition, functional annotation with Ingenuity Pathway Analysis (IPA) identified smoking-correlated genes associated with biological functions in male and female smokers that are directly relevant to well-known smoking related pathologies. However, these relevant biological functions were strikingly overrepresented in female smokers compared to male smokers. IPA network analysis with the functional categories of immune and inflammatory response gene products suggested potential interactions between smoking response and female hormones. Our results demonstrate a striking dichotomy between male and female gene expression responses to smoking. This is the first genome-wide expression study to compare the sex-specific impacts of smoking at a molecular level and suggests a novel potential connection between sex hormone signaling and smoking-induced diseases in female smokers.

## Introduction

Cigarette smoking is the largest single risk factor for premature death in the United States and is responsible for 440,000 deaths every year [1]. Smoking adversely affects almost every human organ and is a predominant cause of many diseases such as cancer, cardiovascular diseases including stroke and heart attack, a range of respiratory diseases, and other severe chronic diseases [2,3]. Smoking also increases the incidence of other adverse health effects such as cataracts, infection and poor wound healing, inflammatory bowel disease and some neurological diseases like Parkinson’s and Alzheimer’s diseases [4,5]. Countless studies have demonstrated that smoking causes lung cancer, and smoking has also been shown to substantially increase the risk of developing cancer of the larynx, pharynx, oral cavity, esophagus, stomach, liver, colon, rectum, pancreas, bladder, kidney, pharynx, nasal cavity, cervix and prostate [6].

Increasing evidence suggests respiratory symptoms vary by sex, smoking habits and age. It has been reported that the health effects of smoking are more serious for women than for men [7]. For instance, women are more vulnerable to cigarette smoke-induced respiratory diseases [8,9]. Smoking also adversely affects the fertility of women, causes early menopause [8] and increases hazards in pregnancy [7]. Cigarette smoking is an established predictor of type-2 diabetes mellitus, and female smokers possess much higher risk of type-2 diabetes mellitus than their male counterparts [10]. Previous studies also suggested that female smokers are more susceptible to tobacco carcinogens [11–13]. The patterns of various types of lung cancer incidence suggest that women have a higher absolute risk for lung cancer than do men of the same age with the same history of smoking [12–16]. In addition to common smoking-induced carcinogenic effects in both sexes, women suffer additional hazards in female-specific cancers such as breast cancer [17], ovarian cancer [18] and cancer of the cervix [7]. A recent study also suggests that smoking increases the risk of colorectal cancers in female compared to male smokers [19].

Sex differences in rates of survival following diagnosis of lung cancer have also been reported. Interestingly, women have been found to have higher survival rates regardless of lung cancer type, stage and therapy [14,20–23]. In addition to lung cancer, women have a higher five-year survival rate than men for the majority of cancers with the exception of bladder cancer, for which women have lower survival [24]. Women’s better survival rate from the majority of smoking-associated cancers has remained a puzzling issue. It is presently unclear whether the basis for this difference is biological, social, or behavioral, and these issues are difficult to resolve through epidemiological studies.

The toxic components of cigarette smoke enter the blood stream through the pulmonary alveoli and are distributed throughout the body. Thus, blood may provide an appropriate biological material in which to study the systemic effects of cigarette smoke exposure. Several studies have investigated gene expression in smokers’ peripheral blood lymphocytes and reported substantial numbers of genes differentially expressed in smokers and non-smokers [25–29]. While these studies have identified a large number of genes apparently responding to smoking, very few genes have been found to overlap among the studies.

Moreover, although many clinical and epidemiological studies have indicated that women are more vulnerable to smoking associated diseases, no genome-wide expression study has investigated the effect of sex on the smoking response. Very recently, Pan et al., [30] compared gene expression profiles of B cells between white female smokers and non-smokers. They reported that over 75% of the smoking responsive genes were down regulated and these down-regulated genes predominantly belonged to functional categories involved with immune responses. However, the expression pattern of this gene set in male smokers is unknown and thus sex-specific differences cannot be determined.

In the present study, we compared gene expression profiles of peripheral blood cells between smokers and non-smokers using Agilent whole-genome microarrays, and distinguished patterns of smoking-related gene expression that differ dramatically between males and females. A large number of the smoking-associated genes identified were directly relevant to well-known smoking-related pathologies, and in females were connected to signaling by estrogen and progesterone. This is the first genome-wide expression study on the sex-specific impact of smoking as reflected in peripheral blood cells, and our findings may begin to suggest molecular connections underlying some of the differences in smoking-related cancer risks and outcomes previously found in epidemiological studies.

## Materials and Methods

### Study population

Healthy smokers and non-smokers were recruited with written informed consent. The study was approved by the Institutional Review Board of Columbia University. A total of 24 males and 24 females consisting of an equal number of smokers (≥1 pack per day) and non-smokers (never smokers) of each sex participated in this study. Characteristics including age, sex, and details of smoking habit were provided by the subjects at the time of enrollment. All participants also reported were not under any therapeutic drug or no record of recent radio diagnostic examination. Characteristics of the study population are summarized in Table 1.

### Sample collection and RNA preparation

Peripheral blood from smoker and non-smoker volunteers was drawn into 0.105 mol/l sodium citrate Vacutainer tubes (Becton Dickinson and Company, Franklin Lakes, NJ). RNA was prepared using the PerfectPure RNA blood kit (PerfectPure, Gaithersburg, MD) as recommended by the manufacturer, followed by GLOBINclear (Ambion Inc., Austin, TX) treatment to further reduce levels of both α- and β-globin. The RNA was quantified using a NanoDrop-1000 spectrophotometer, and quality was monitored with the Agilent 2100 Bioanalyzer (Agilent Technologies, Santa Clara, CA). All RNA samples had RNA integrity numbers between 7.8 and 9.1 (mean, 8.4). RNA was stored at −80°C until use.

### Microarray hybridization and expression profiling

Cyanine-3 (Cy3)-labeled cRNA was prepared from 0.5 μg input RNA using Agilent Technologies’ One-Color Quick Amp labeling kit according to the manufacturer’s instructions, followed by purification of cRNA by RNAeasy column (QIAGEN, Valencia, CA). Cy3-dye incorporation and yield of cRNA was checked with a NanoDrop ND-1000 spectrophotometer. The specific activity of all cRNA samples ranged from 10.98 to 20.02 (mean 14.94). cRNA (1.65 mg) was fragmented and hybridized to Agilent’s whole genome microarrays (G4112A) at 65°C for 17 hr with rotation, followed by washing according to the manufacturer’s recommendations. Microarray slides were scanned immediately with the Agilent scanner (G2404B) using the recommended settings and the images were analyzed with Feature Extraction 9.1 (Agilent Technologies) using default parameters.

### Data analysis

Background corrected hybridization intensities were imported into BRB-ArrayTools Version 3.8.0 beta [31], log2 transformed and normalized using the median over the entire array. Features that were non-uniform outliers or not significantly above background intensity in 25% or more of the samples, or that did not change at least 1.5-fold from the median value in at least 20% of the experiments were filtered out. This resulted in 16,548 features that were used in subsequent analyses. The microarray data is available online on the NCBI’s Gene Expression Omnibus using accession number GSE47415.

BRB class comparison was applied to identify genes that were differentially expressed between smokers and non-smokers using a two-sample Student t-test. Genes with p-values of p<0.005 were considered statistically significant. The false discovery rate (FDR) was also estimated for each gene using the method of Benjamini and Hochberg [32] to control false positive results. The 2-way mixed model ANOVA from BRB-ArrayTools Plugins was used to identify smoking-responsive genes that were affected by sex.

Multidimensional scaling (MDS) was used in BRB-ArrayTools to visualize differences between groups at the transcriptional level based on differentially expressed sets of genes using the Euclidian distance metric to compute a distance matrix and the principal components of the gene expression signature. In the output, each sample is represented by a single point and the distance between any two points indicates the overall similarity of the two represented samples. In addition, we executed hierarchical clustering analysis to generate a heat map image of the expression of genes differentially expressed between smokers and non-smokers using the Euclidean distance matrix.

### Pathway and networking analysis by ingenuity pathway analysis (IPA)

The significantly differentially expressed genes in male and female smokers were imported into IPA version 7.6 (Ingenuity^®^ Systems, www.ingenuity.com). Following collation, there were 131 and 191 transcripts in males and females, respectively that mapped to known genes in IPA. We first used the IPA biomarker analysis workflow to identify promising molecular biomarker candidates from the dataset. The IPA-Biomarker filter mapped 124 and 175 biomarker candidate genes in male and female smokers, respectively. We next performed IPA core-analysis in the context of pathways and networks, biological function and/or diseases. The right-tailed Fisher’s exact test was applied to calculate the p-value ascertaining the probability that each biological function and/or disease assigned to that dataset was due to chance alone.

We used network analysis to determine whether immune and inflammatory response gene products in female smokers were connected at the molecular network level based on connectivity information in the IPA Knowledge Base. We added molecules suggested by the IPA “pathway explorer” in order to connect molecules of interest. Priority was given to those molecules with a high degree of connectivity within the pathway rather than molecules with many connections to molecules not on the pathway.

### Gene set enrichment analysis (GSEA)

We used gene set enrichment analysis (GSEA) for interpreting smoking induced microarray data using the Broad Institute’s GSEA software [33]. GSEA, using the Kolmogorov-Smirnov statistic, incorporates biological knowledge into analysis to identify enrichment of biological functional categories in sets of ranked differentially expressed genes from genome-wide mRNA expression data sets. The GSEA calculates an enrichment score (ES) reflecting the degree a gene set is overrepresented by member genes ranking at the top or bottom of the ranked gene list. The statistical significance of the ES is estimated using a permutation test (p-value) with false discovery rate (FDR) correction for multiple hypothesis testing. We ran 1,000 permutations for each analysis, and used the criteria of nominal p-value<5% and FDR<25% as the statistical cutoff for all analyses. All genes that passed the BRB filtering criteria (above) were imported into the GSEA tool and the data were analyzed according to the recommendations in the GSEA users’ manual.

## Results

### Differential gene expression in smokers’ blood

**Figure 1:**
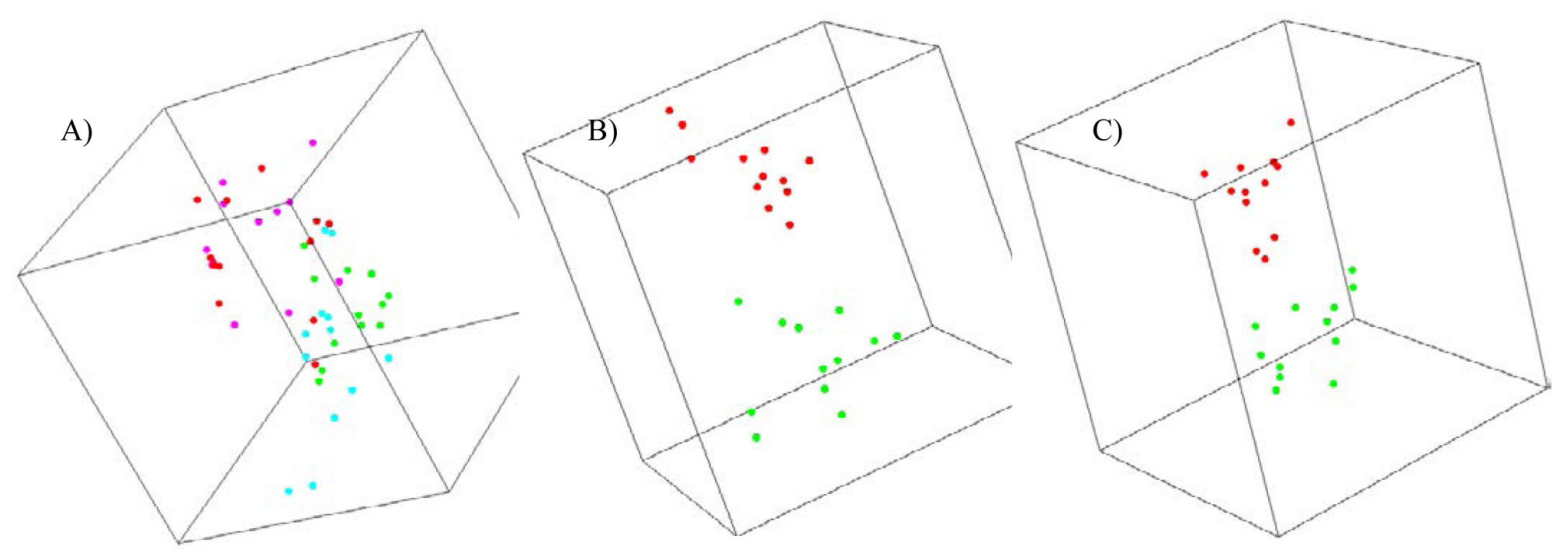
Multidimensional scaling plot summarizing gene expression differences between smokers and non-smokers. Each point represents an individual sample, and the distance between two points reflects the overall similarity in expression of the selected set of genes in those two samples. A) Separation of smokers and non-smokers according to 300 genes differentially expressed between all smokers and non-smokers, irrespective of their sex. The points are colored as red (male non-smoker), pink (female non-smoker), green (male smoker) and cyan (female smoker). B) Separation of male smokers (red) and non-smokers (green) using a set of 175 genes differentially expressed in male smokers and non-smokers. C) Separation of female smokers (red) and non-smokers (green) using a set of 247 genes differentially expressed in female smokers and non-smokers.

Global gene expression was measured in peripheral white blood cells of 48 donors, comprising equal numbers of smokers and non-smokers from each sex. Agilent whole genome microarrays were hybridized using the one-color protocol to identify genes differentially expressed between smokers and non-smokers. We used the class comparison feature of BRB-ArrayTools to identify genes that were differentially expressed between smokers and non-smokers. We identified 300 genes with significantly different expression (p<0.005) (SI Table 2), of which 170 genes (57%) were up-regulated and 130 genes (43%) were down-regulated in smokers. Visualizing the expression of this set of genes by multi-dimensional scaling (MDS) (Figure 1A) reveals a trend of separation of the samples based on smoking status, although the separation is not distinct for all samples, suggesting variability between individuals. The visual trend appears slightly stronger between female smokers and non-smokers than between male smokers and non-smokers.

### Sex-specific smoking signatures

We next investigated the possibility of sex specificity of smoking signatures in males and females. We first performed a class comparison for differentially expressed genes between smokers and non-smokers using only the 24 male donors. This analysis identified 175 genes significantly differentially expressed between male smokers and non-smokers (p<0.005) (SI Table 3). Of the 175 genes differentially expressed, 125 genes (71%) were up-regulated and 50 genes (29%) were down-regulated. This set of 175 genes showed a clear separation between samples from male smokers and non-smokers when visualized by MDS (Figure 1B).

We next used the same analyses to look for altered gene expression between female smokers and non-smokers. In this case, class comparison identified 237 genes as differentially expressed (p<0.005) between female smokers and non-smokers (SI Table 4). Among the 237 potential smoking-modulated genes identified in female smokers, 91 genes (38%) were up-regulated and 146 genes (62%) were down-regulated. Expression of this set of genes visualized by MDS (Figure 1C) again distinctly separated smokers from non-smokers, in this case among female donors. When we attempted to separate smoker and non-smoker samples of each sex using the differentially expressed genes identified from the opposite sex, MDS failed to discriminate between smokers and non-smokers both in males and females (not shown), further suggesting a strong sex-specificity in the gene expression response to smoking.

Although a substantial number of genes were found to be differentially expressed in both male and female smokers, a divergent pattern of expression was apparent on the basis of the number of genes up- or down-regulated in male and female smokers. The intersection of male and female gene sets yielded only 13 genes (3%) that responded significantly in both sexes (Table 2).

To investigate further if cigarette smoking has any significant interaction with sex, we applied BRB-ArrayTools’ 2-way mixed model ANOVA. This analysis identified eighty genes with smoking responses modified by sex (p<0.005) (SI Table 5). Interestingly 44% of these genes were also identified as differentially expressed in the female smokers and 14% in the male smokers.

### Functional network analysis

**Figure 2:**
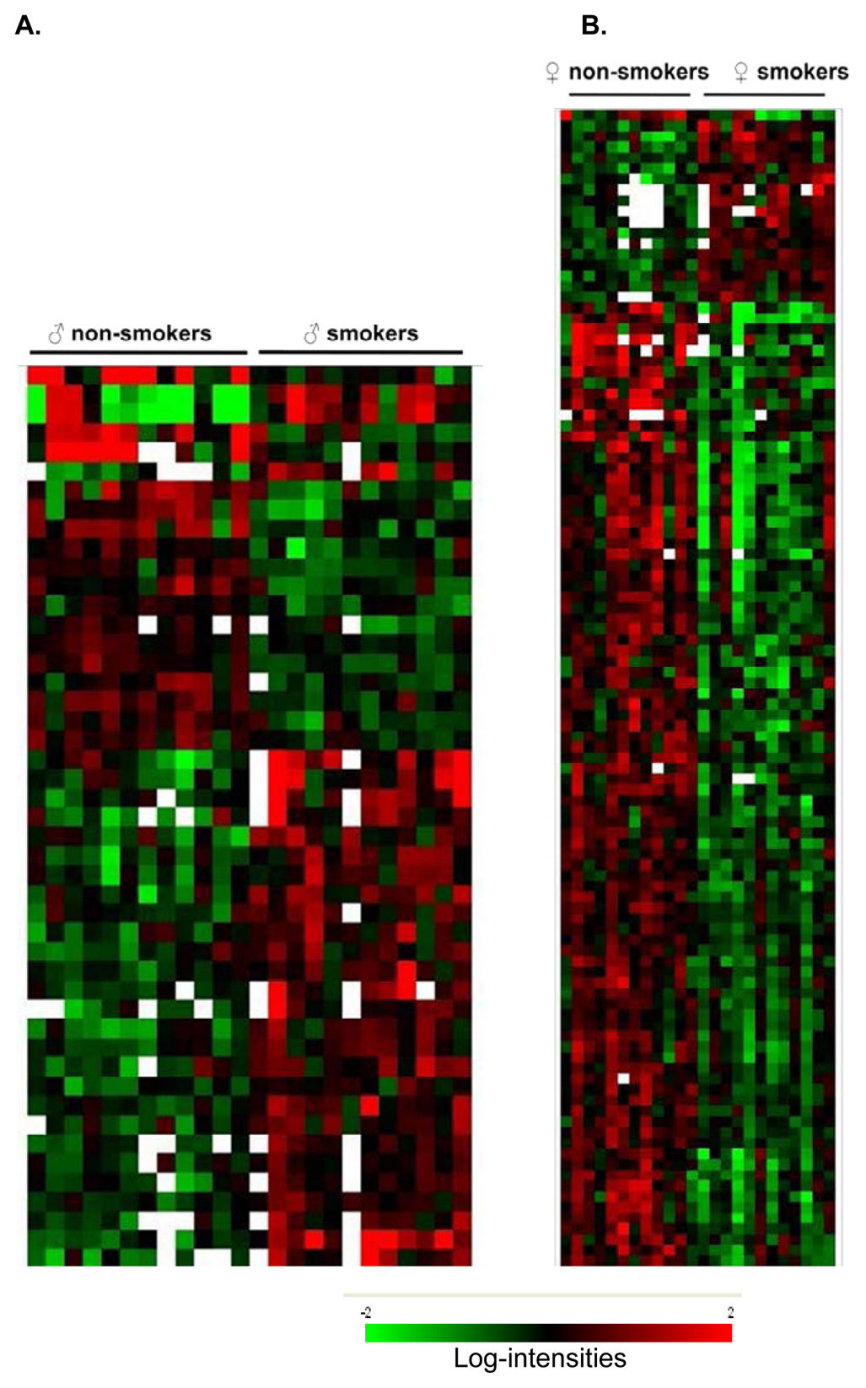
Hierarchical clustering of smoking correlated genes corresponding to IPA functional categories in the context of pathways and networks, biological function and/or diseases (presented in Table 2). A supervised average linkage clustering of 47 genes in male (A) and 111 genes in female (B) smokers is presented. The annotation of all genes in clustered order is presented in SI Table 6.

To further assess the sex specific smoking response, we performed pathway analysis with the gene sets differentially expressed in male and female smokers using Ingenuity Pathway Analysis (IPA), version 7.6. First, we identified overrepresentation of smoking-affected genes within known functional categories (Table 3). Although many functional categories were significantly affected in both sexes, some were unique to female or male smokers, suggesting that biological consequences of smoking could be very different in males and females. In female smokers, the most significant disease categories correlated with neurological disease, infectious disease, inflammatory disease, cardiovascular disease, immunological disease, hematopoiesis, respiratory disease, diabetes mellitus and cancer. In male smokers, the highly significant disease categories were cancer, diabetes mellitus, neurological diseases and cardiovascular diseases. The expression pattern of male and female smoking correlated genes linked to biological function and/or diseases is presented by heat-map in Figure 2.

The over-represented biological function categories also differed extensively between male and female smokers (Table 3). Further, in female smokers, most genes in the biological function categories were down regulated, whereas in male smokers the majority of the genes involved in these categories were up regulated. In addition, some biological function categories, such as genes involved in embryonic development, cellular compromise, free radical scavenging and DNA repair, were only significant in female smokers, and the genes within these functional categories were overwhelmingly down-regulated in female smokers.

There was also high over-representation of canonical pathway categories of smoking correlated genes in female smokers that were related well to known smoking pathologies, shown in SI Table 7. The most striking of these canonical pathway categories are xenobiotic metabolism signaling, actin metabolism signaling, clathrin-mediated signaling, eicosanoid signaling, thrombin signaling, tight junction signaling, molecular mechanism of cancer and natural killer cell signaling. Of specific interest, there were 6 genes involved in metabolism of xenobiotics by Cytochrome 450 in our female smokers data set, three of which (AKR1C3, DHRS2, GSTA2) were negatively correlated and three others (UGT1A6, CYP4F2, CYP4F12) were positively correlated. Smoking introduces a large number of xenobiotics into a smoker’s body and Cytochrome P-450 enzymes have been indicated to detoxify tobacco carcinogens [34].

### Network analysis

**Figure 3:**
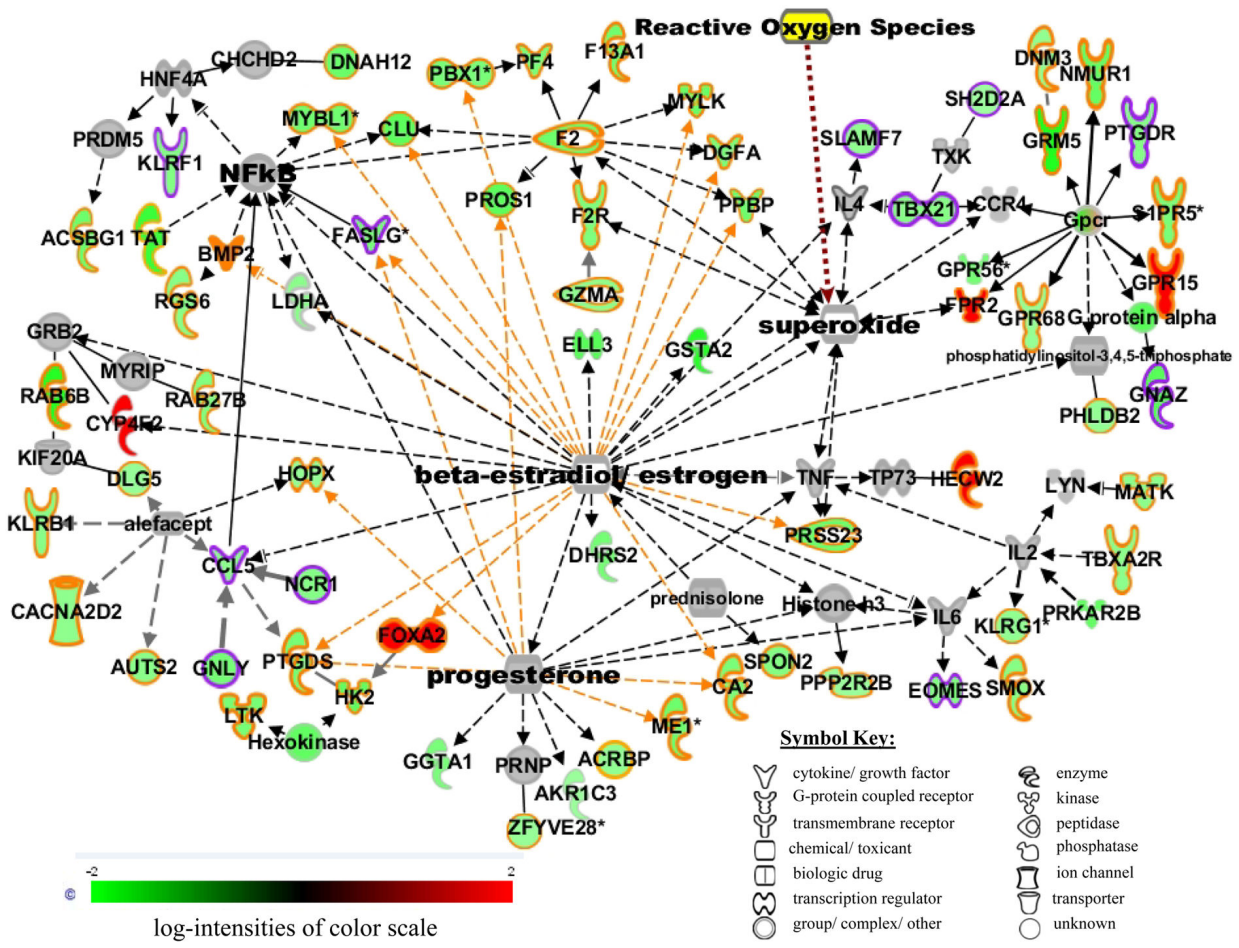
Gene product interaction network of immune and inflammatory responsive genes associated with smoking in females generated from the information in the Ingenuity knowledge base, version 7.6. Genes or gene products are represented as nodes, and the biological relationship between two nodes is represented as an edge (line). Solid lines represent direct relationship and dashed line represents indirect relationship between nodes. The intensity of node color indicates the degree of up- (red) or down- (green) regulation in smokers. Grey nodes represent molecules not altered in smokers added by Ingenuity to show network connections. The yellow node is a cigarette smoke toxicant manually added to the network. The shape of each node indicates the gene product’s functional class as shown in the key. The genes involved in immune/inflammatory responses are highlighted in yellow, and those involved both in immune/inflammatory responses and the NK cell signaling pathway are highlighted in purple. Immune/inflammatory genes known to be influenced by female hormones (progesterone and estrogen) are linked with yellow edges.

The functional categories of immune and inflammatory responses/diseases differed widely between smoking-responsive genes in male and female smokers (Table 3). We added molecules suggested by the IPA “pathway explorer” in order to connect molecules of interest. The resulting network connected 64 genes associated with immune and inflammatory response in female smokers into a single network (Figure 3) that suggested potential interactions between smoking response and female hormones. It was also of interest to explore the immune and inflammatory response gene products modulated in response to male smoking in comparison with the female network (Figure 3). We found that only six of the genes in the female network were differentially expressed in male smokers, four of which were up-regulated (RGS6, ELL3, TBXA2R and GRM5) and two down-regulated (RAB6B and GPR15).

### Gene set enrichment analysis

**Figure 4:**
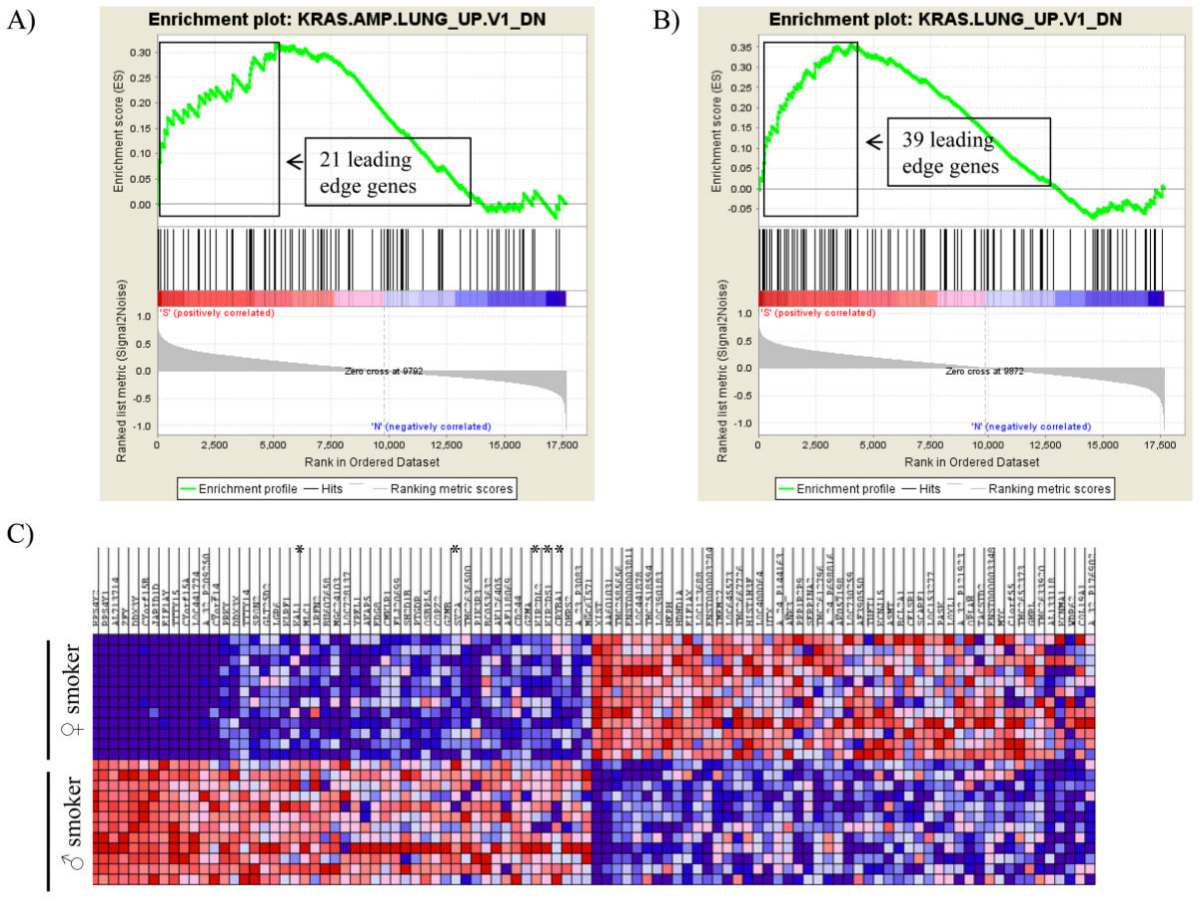
Enrichment of the over-expressing oncogenic form of KRAS, most commonly mutated oncogenes in lung cancer, in male non-smokers vs smokers (A) and female non-smokers vs smokers (B). In response to smoking, 21 genes associated with KRAS oncogene were overexpressing in male smokers and that of 41 genes in female smokers. C) heat map showing the top 50 genes/features representing oncogenic signature gene-sets over-represented in male and female smokers (red=up-regulated, white=average expression, blue=down-regulated). Natural killer cell-mediated cytotoxicity genes down-regulated in female smokers are marked with asterisks along the right edge.

Smoking undeniably causes a different pattern of elevated cancer risk in females and many clinical studies have reported that women have a higher absolute risk of smoking induced cancers [12–16,34], although women have been found to have better survival from the majority of smoking-associated cancers [14,20–23]. We used gene set enrichment analysis (GSEA) [32] to confirm and explore concordant differences between the two biological states of male and female smoking against oncogenic signature gene sets. The GSEA analyses identified 12 significant gene sets as a result of male smoking and 14 gene sets for female smoking at p-value<5% (SI Table 8A and 8B, respectively). Of the enrichment gene sets in male and female smokers only the over-expressing oncogenic form of KRAS.LUNG was common to both with 21 “leading edge genes” in male smokers and 39 “leading edge genes” in female smokers (Figure 4a and 4b). Interestingly, the KRAS. PROSTATE gene set was up-regulated in male smokers and the BRCA1 gene set was down-regulated in female smokers. The top oncogenic signatures identified from male smoking include tumor suppressor genes (PTEN and RB1), colon cancer gene sets (CTIP and SNF5), skin tumor progression protein (ATF2), oncogenic signatures KRAS-600-LUNG and E2F3 pathway genes. The top female smoking enrichment oncogenic signatures are colorectal carcinoma genes sets (KRAS.600), neoplasias of kidney (KRAS.KIDNEY), notch signaling pathway gene set (NOTCH), anaplastic lymphoma kinase (ALK), polycomb ring finger oncogene (BMI1), and activating transcription factor 2 (ATF2) etc. The top 50 genes/features representing oncogenic signature gene-sets over-represented in male and female smokers are displayed as a heatmap in Fig. 4 c. Natural killer cell-mediated cytotoxicity genes play an important role in rejection of tumors, and are overwhelmingly down-regulated in female smokers (Figure 4c).

## Discussion

The effects of cigarette smoking on human health are serious and in many cases, deadly. Cohort epidemiological studies have shown that women are more vulnerable to cigarette-smoking induced diseases; however, the molecular basis of these differences has remained unclear. In this study human peripheral blood was used to explore sex-specific smoking induced differential gene expression between smokers and non-smokers. Cigarette smoke toxicants enter the body through the pulmonary alveoli and are directly absorbed and distributed throughout all tissues. Blood cells are not only one of the most accessible tissues for gene expression analysis, they also have been shown to be an excellent tissue type to study environmental exposures, like cigarette smoke [29] and radiation exposure [35]. Blood cells have the potential to reflect systemic damage occurring in different organs and tissues as a result of smoking. Unlike previous studies in which smoking induced gene expression was measured in peripheral blood lymphocytes [28–30], we used peripheral blood leukocytes to identify smoking related changes in gene expression. Thus many of the genes we found to be differentially expressed in the peripheral blood of smokers compared to non-smokers have not been reported by other studies.

The male and female smoking-correlated genes associated with biological function and/or diseases identified in this study are directly relevant to well-known smoking related pathologies. The results include strong involvement of a range of smoking-correlated diseases such as cancer, neurological disease, immune response, inflammatory diseases, cardiovascular disease, respiratory disease, hematological disease, cell death and proliferation, cellular development and cell-to-cell signaling, and xenobiotic metabolism. However, the extent of these relevant biological function and/or diseases, the number of correlated genes and their expression patterns were strikingly over-represented in female smokers (Table 3). Our observations are consistent with many clinical studies demonstrating that the health consequences of smoking for women are worse than for men [7–10,19,36].

Cigarette smoking is an established risk factor for many cancers, not only at the site of contact but also throughout the body. We identified sets of smoking correlated genes in both male and female smokers with documented associations with various cancers, consistent with risks observed in epidemiological studies. One remarkable finding of this study was the altered expression of genes corresponding to sex specific cancers, hyperplasia of prostate in male smokers and epithelial ovarian cancer in female smokers. Epidemiological studies have suggested that smoking increases the risk of ovarian [18] and prostate [37] cancers in female and male smokers, respectively. Another important finding was significance of colorectal cancer in female smokers. It has been suggested that smoking increases the risk of colorectal cancers to a greater extent in female compared to male smokers [19]. Furthermore, a higher number of smoking-induced alterations in expression of cancer related genes in female smokers (Table 3) are consistent with the increased susceptibility of female smokers to tobacco carcinogens compared to male smokers.

Gene set enrichment analysis (GSEA) with oncogenic signature gene sets further substantiated the gene expression differences underlying the female smokers’ heightened susceptibility to tobacco carcinogens. GSEA identified a large number of oncogenic pathway gene-sets that were significantly altered in female smokers compared to male smokers. Our data corroborate the findings of other investigators suggesting that women are more susceptible than men to the ill effects of the carcinogens in tobacco and tobacco smoke [11,12,15,16]. Among various functional groups exclusively significant in female smokers are DNA repair, xenobiotic metabolism, free radical scavenging and NK cells cytotoxicity, and the genes corresponding to these functional categories are overwhelming down-regulated in female smokers. NK cell cytotoxicity involves defense against foreign cells and plays an important role in rejection of tumors. In a murine lung metastasis tumor model, NK cell tumor immune surveillance has been shown to decrease in response to cigarette smoke exposure [38], consistent with our findings in female smokers. Down-regulation of oxidant scavengers may contribute to the down-regulation of DNA repair genes we found in female smokers. In our study, and consistent with epidemiological studies [7,12,14,17,19], down-regulation of oxidant scavengers and oxidative damage repair genes in female smokers may contribute to increased risk of cancers compared to non-smokers, or compared to male smokers, where this pattern of gene expression was not found.

Although female smokers have a higher absolute risk of developing cancers than their male counterparts, women’s better survival rate from smoking-associated cancers [12,14,24] has remained a puzzling issue and no molecular mechanism has been proposed to date to explain it. We have found several broad differences in the smoking-related pattern of gene expression that are highly intriguing in this regard. For example, we identified three genes of the K-ras oncogene family, of which two were up-regulated in male smokers (RAB6B, RAB42) and two were down-regulated in female smokers (RAB6B, RAB27B). In addition, two genes for cytochrome P-450 enzymes (CYP4F2, CYP4F12) were found to be up-regulated in female smokers. It has been reported that cytochrome P-450 enzymes may be able to detoxify tobacco carcinogens by repairing smoking-induced DNA adducts [39]. It has been reported that there is a high variability among different ethnic groups in the activity of the cytochrome P-450 enzymes (CYP) due to genetic and environmental factors, like smoking and alcohol consumption [40]. Polymorphic CYP genes are capable of creating differences in the ability to metabolize, detoxify or activate xenobiotic chemicals. Several studies have demonstrated that certain polymorphic CYP genes, in association with smoking and alcohol consumption, are involved in the development of certain cancers [41–43]. Therefore, further studies recruiting larger population of male and female smokers and non-smokers including different ethnic groups will be necessary to determine the consequences of active smoking on genetic variation and genders. The GSEA analysis also indicated that the gene-set involved in the TP53 tumor suppressor pathway was up-regulated in female smokers but down-regulated in male smokers. In addition, tumor suppressor pathway, the PTEN and RB1gene-sets, were significantly repressed in male smokers. It is currently unknown to what extent any of these genes or pathways may play a role in changing the balance of survival in cancer. These very different patterns of gene expression could, however, provide a basis for further research with male and female patients with smoking-associated cancers to help unravel the mechanisms involved in the different relative survival in smoking associated cancers.

Expression of genes within the functional categories of immune and inflammatory responses/diseases also differed widely between male and female smokers. Compared to male smokers, a large number of genes corresponding to immune alteration and suppression were altered in female smokers. In a recent study, Pan et al., [30] demonstrated that the overwhelming majority of smoking correlated genes were down-regulated in peripheral B-cells of female smokers, and that these down-regulated genes predominantly belonged to functional categories involved with immune responses. The overwhelming down-regulation of natural killer (NK) cell signaling pathway genes, involved in cytotoxicity and cytokine secretion, in response to smoking among females might have an impact associated with immune and inflammatory diseases. This wide range of negative influence on the immune system in female smokers might also have been related to the over-representation of functional groupings relevant to neurological, infectious, cardiovascular, hematological, renal and urological, diabetes and respiratory diseases in female smokers. Previous clinical studies have suggested that women may be more susceptible than men to cigarette smoke-induced respiratory diseases [8,9] and possess much higher risk of type-2 diabetes mellitus than their male counterparts [10]. In addition a large set of genes related to inflammatory diseases including bowel disease, Crohn’s disease, digestive disorders and arthritis were highly significant in female smokers. A clinical study partly corroborated our findings with the demonstration that smoking adversely affects Crohn’s colitis by sex, with women having more disadvantage than men [44].

Since all these studies suggest the importance of immune and inflammatory response in the female smoking response, we constructed a network of immune and inflammatory response affected by smoking in females based on connectivity information of gene/gene product interactions from the IPA Knowledge Base (Figure 3). This network includes 70 genes significantly correlated with female smoking, 64 with known involvement in immune/inflammatory responses (highlighted in yellow in Figure 3) or in both immune/inflammatory and NK cell signaling pathways (highlighted in pink in Figure 3). This network also connects to multifunctional pathways such as NFκB, cytokine receptors (IL2, IL6), Gpcr and free radicals. The network also clearly displays a major interaction with two female hormones, progesterone and estrogen, with many of the smoking-responsive immune and inflammatory genes, as well as portions of the sub-networks of some essential signaling pathway molecules. This network suggests a potential regulatory link between the immune and inflammatory genes differentially expressed in smokers and female hormones, as well as functional pathways linked to carcinogenesis and inflammatory diseases.

The present study was carried out with a modest number of smokers and non-smokers with a broad range of ages using peripheral blood leukocytes. Future investigation of smoking effects on different lymphocyte populations in both sexes may provide more precise information of the impact of sex on the physiological response to smoking. The present study suggests genes associated with immune functions are severely affected in female smokers. Thus, it may be informative to examine smoking impacts specifically in B cells in both male and female smokers. To further examine the impact of sex on the response to smoking, our results suggest that it will be essential to study sex-specific gene expression changes in female smokers in different age groups. If the gene expression response to smoking is, indeed, influenced greatly by interactions with female hormones, the smoking-induced gene expression responses seen in pre- and post- menopausal women should differ significantly. Some recent studies have indicated a link between menopausal hormone treatment therapy and increased lung cancer risk in females, and female smokers having hormone therapy possessed a particular high risk of mortality from lung cancer [45,46]. This further underlines the importance of investigating potential interactions between female sex hormones and smoking as a step toward understanding the molecular mechanisms underlying the observed sex-based differences in smoking-related cancer incidence and survival. Several recent works have demonstrated that some common genes were modulated from alcohol abuse and tobacco smoking in animal models and in human subjects [47,48]. Thus, it will be crucial to record detail life style of the study population in future study.

## Conclusion

This is the first genome-wide expression study to compare the sex-specific impact of active smoking in vivo. Using defined functional network analyses we have identified sets of altered genes related to a large number of smoking-induced pathologies in both sexes. Based on defined functional relationships, we suggested a potential novel connection between sex hormone signaling and smoking-induced diseases in female smokers (Figure 3). However, further studies with larger populations of smokers with different smoking habits, non-smokers, and previous smokers, both in good health and with smoking associated disease and other potential confounding factors such as differences in age, lung function, body mass index, race, etc., will be necessary to understand the impact of smoking at the molecular level more clearly.

## Supplementary Material

SI Table 1

SI Table 2

SI Table 3

SI Table 4

SI Table 5

SI Table 6

SI Table 7

SI Table 8

## Figures and Tables

**Table 1:** Summary of study population characteristics.

Characteristic	Smokers (n=24)		Non-smokers (n=24)	
	Women (n=12)	Men (n=12)	Women (n=12)	Men (n=12)
Age (mean)	39.5	42.5	33.5	38.5
Pack/day of smoking (mean)	1.21	1.21		
Years smoked (mean)	19.83	21.67		
Age at start of smoking (mean)	18.67	20.83		

Note: Age and smoking status of the study population presented were not significant (p<0.05). The level of significance of age between smokers and non-smokers of both sexes, the p-vale of smoking status depending on years smoked, number of cigarettes smoked and the age of starting smoking between male and female smokers are presented in SI Table 1.

**Table 2:** Genes with significant differential expression in both male and female smokers showing the relative fold-change associated with smoking.

Probe ID	Gene Symbol	Male fold-change	Female fold-change
A_24_P83899	GRM5	0.36	0.3
A_23_P6943	GPR15	1.96	2.14
A_24_P47988	ELL3	0.31	0.4
A_23_P353524	IVL	1.6	1.96
A_24_P83799	ANKRD33B	0.49	0.43
A_23_P90357	TBXA2R	0.63	0.59
A_23_P358709	AHRR	5.11	5.11
A_23_P205666	RGS6	0.69	0.56
A_23_P109974	RAB6B	2.58	0.31
A_23_P162314	DHH	0.42	0.49
A_24_P933688	NAV2	2.78	1.66
A_23_P312752	KCNJ13	2.24	1.84
A_24_P339560	SIGLEC11	2.26	2.32

**Table 3:** IPA functional analysis of sex-specific smoking correlated genes differentially expressed in smokers’ peripheral blood cells.

			Female			Male	
	Disease		p-value^a^	#Genes b		p-value	#Genes
**a)**	Neurological disease		6.25E-8 - 5.81E-3		54(8)	4.06E-3 - 2.41E-2	8(2)
	Rett syndrome	2.73E-8		9			
	Psychological disorder	5.03E-5		26			
	Mood disorder	8.94E-5		20			
	Bipolar affective disorder 7.19E-5		18				
	Taupathy	1.58E-3		14			
	Encepahlopathy	1.79E-5		10			
	Neuropathy	2.04E-3		17			
	Neurodegenerative disease	2.34E-3		14			
	Alzheimer’s disease	3.72E-3		13			
	Neurological disorder	5.81E-3		52	2.41E-2	8	
**b)**	Infectious disease		9.17E-7		8(0)	2.76E-2	1(0)
**c)**	Inflammatory disease/disorder		1.49E-5 - 1.66E-3		40(6)	NS^c^	
	Inflammatory bowel disease	1.42E-4		20			
	Crohn’s disease	1.42E-4		19			
	Arthritis	4.89E-4		24			
	Digestive system disorder	1.47E-3		20			
	Genetic disorder of heart	6.69E-5		3			
**d)**	Cardiovascular disease		2.25E-5 - 9.47E-03		32(3)	5.58E-3 - 6.45E-2	13(9)
	Atherosclerosis	2.25E-5		21	3.99E-2	7	
	Thrombosis	2.26E-4		5			
	Coronary artery disease	5.96E-4		17	6.54E-2	6	
	Cardiovascular disorder	1.17E-3		30	1.96E-2	13	
	Myocardial infraction		6.65E-3		4		
	Cardiac infraction		9.47E-3		4	3.30E-2	1
**i)**	Immunological diseases		1.22E-4 - 5.09E-3		29(4)	2.21E-2	2(2)
	Rheumatoid arthritis		3.31E-3		21		
	Autoimmune disease		5.09E-3		27		
**j)**	Hematopoiesis		2.65E-4		13(0)	1.01E-2	3(1)
**k)**	Respiratory disease		3.99E-3		11(2)	3.32E-2	2(1)
**l)**	Cancer		5.28E-3 - 1.47E-2		19(5)	3.07E-5 - 3.84E-2	14(6)
	Hyperplasia of prostrate	NS			3.07E-5	5	
	Hyperproliferation	NS			3.29E-2	5	
	Colorectal cancer	8.32E-3		13	NS		
	Epithelial ovarian cancer 9.18E-3		4	NS			
	Transformation	1.22E-2		6	NS		
**m)**	Diabetes mellitus	8.31E-3		24(2)	3.01E-3	11(6)	
**n)**	Renal and urological disorder		9.47E-3		10(0)	5.58E-3	1(1)
**o)**	Organismal injury and abnormalities		9.47E-3		9(0)	5.58E-3	3(1)
**p)**	Connective tissue & muscular disorder		9.47E-3		25(1)	NS	

a The p-value is calculated using the right-tailed Fisher Exact Test.

b The number of smoking-correlated genes in each category of functional assignments is presented. The number of genes up-regulated in each category is shown in parentheses.

c NS: Not Significant.
