# Supplementary material for: Differential Effect of Active Smoking on Gene Expression in Male and Female Smokers
Source: J Carcinog Mutagen. Author manuscript; Available in PMC 2015 Jan 22. (PMC4303254; doi:10.4172/2157-2518.1000198)
Supplement: SI Table 7 [file NIHMS653150-supplement-SI_Table_7.docx]

**SI Table 6.** Smoking correlated canonical pathway categories over-represented in smokers including number of genes involved in the corresponding category.

**Female Male**

1. G-protein coupled receptor signaling 11 4
2. Actin metabolism signaling 6 2
3. Xenobiotic metabolism signaling 5
4. Metabolism of xenobiotic by Cytochrome 450 6
5. Natural killer cell signaling 3
6. Clathrin-mediated signaling 5 2
7. Eicosanoid signaling 5
8. Thrombin signaling 5
9. Tight junction signaling 5
10. Molecular mechanism of cancer 4 2
11. Bladder cancer signaling N 2
12. FGF signaling N 2
13. Hepatitic fibrosis 4
14. Arachidonic acid metabolism 4
15. Ga 12/13 signaling 4
16. Glycolysis/gluconeogenesis 4
17. Protein kinase A signaling 4 2
18. Cellular effects of slidenafil (Viagra) 4
19. Axonal guidance signaling 4
20. Coagulation system 4
21. AMPK signaling 3
22. Aryl hydrocarbon receptor signaling 3
23. Dopamine receptor signaling 3
24. CREB signaling in necrosis 3
25. LPS/IL-1 mediated inhibition of RXR function 3
26. Calcium signaling 3
27. P7056 signaling 3
28. Cardiac hypertrophy signaling 3
29. Starch and sucrose metabolism 3
30. Synaptic long term depression 3
31. Corticotrophin releasing hormone signaling 3
32. Intrinsic prothrombin activation pathway 3
33. Extrinsic prothrombin activation pathway 3
34. TR/RXR activation 3
35. G beta gamma signaling 2
36. Cdc 42 signaling 2
37. Sphingsine-phosphate signaling 2
38. Glucocorticoid receptor signaling 2
39. CCR5 signaling in macrophages 2
40. Human embryonic stem cell pluripotency 2
41. GNRH signaling 2
42. Androgen signaling 2
43. CXCR4 signaling 2
44. CDK5 signaling 2
45. Ephrin receptor signaling 2
46. IL-1 signaling 2
47. Breast cancer regulation by stathmin 1 2
48. Glutamate receptor signaling 2
49. G-protein coupled receptor signaling 2
50. ILK signaling 2
51. Creamide signaling 2
52. ERL/MAPK signaling 2
53. Acute phase response signaling 2
54. Neuropathic pain signaling 2
55. Huntington’s disease signaling 2
56. Cardiac beta-adrenergic signaling 2
57. RhoA signaling 2
58. Regulation of actin-based motility by Rho 2
59. Renin-angiotensin signaling 2
60. VDR/RXR activation 2
61. PRX/RXR activation 2
62. Histidine metabolism 2
63. Tyrosine metabolism 2
64. Pyrimidine metabolism 2
65. Purine metabolism 2
66. Glycolipid metabolism 2
67. Arginine and proline metabolism 2
68. Pyruvate metabolism 2
69. Relaxin metabolism 2
70. Fatty acid metabolism 2
71. Galactose metabolism 2
72. Aminosugars metabolism 2
73. Fructose and mannose metabolism 2
